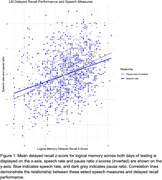# Logical Memory Performance and Speech Metrics in the California Cognitive Assessment Battery

**DOI:** 10.1002/alz70857_106953

**Published:** 2025-12-25

**Authors:** Kathleen Hall, Michael Blank, Kristin Geraci, Isabella Jaramillo, Omar Kahly, Miranda Miranda, Peter Pebler, David K Johnson, David L. Woods

**Affiliations:** ^1^ Neurobehavioral Systems, Inc, Berkeley, CA, USA; ^2^ UC Davis Alzheimer's Disease Center, Walnut Creek, CA, USA

## Abstract

**Background:**

Logical memory (story recall) tasks are frequently used to assess episodic memory and language abilities in older participants to assist in detecting mild cognitive impairment (MCI) and Alzheimer's disease (AD), [1]. Here we describe a logical memory encoding and recall (LM) task from the California Cognitive Assessment Battery (CCAB) [2]. Administration and scoring of this task is automated, and allows for the automatic extraction of speech and language biomarkers (SLBs).

**Method:**

PARTICIPANTS: 772 participants (55% female, 66.5 ± 8.5 years) completed the CCAB logical memory task in their homes during normative data collection. Participants underwent two days of identical testing.

TECHNOLOGY: CCAB LM administration is fully automated, with instructions delivered using text‐to‐speech and responses transcribed and timestamped using consensus automatic speech recognition (CASR). Participants are remotely monitored through the CCAB browser‐based interface, which provides an A/V feed and videochat capabilities. Acoustic and phonetic measures are quantified from high quality (48 kHz, 24‐bit) recordings obtained with a head‐mounted microphone while linguistic SLBs were quantified from CASR generated transcripts.

TASK: Participants heard a novel story, and were then asked to immediately recall the story followed, with delayed recall occurring ∼30 min later. Responses were automatically scored for total match count against a scoring set of 49 keyword elements.

**Result:**

Analysis of immediate and delayed recall of the LM tasks revealed excellent test‐retest values for total match count (*r* = .77 and r = .79, respectively) with significant effects of vocabulary***, age***, gender**, education**, and race* on both immediate and delayed recall. SLBs such as speech rate and pause ratio showed good reliability (*r* = .61, r = .58). and significant correlations (*r* = .32***, r = .42***) with delayed recall performance.

**Conclusion:**

The CCAB's logical memory tasks provide automated objective analysis of recall performance and SLBs to assess episodic verbal memory and communication abilities in older adults.

**References**

[1] Wechsler, D. (1945). Wechsler memory scale.

[2] Woods, D., Pebler, P., Johnson, D. K., Herron, T., Hall, K., Blank, M., … Baldo, J. (2024). The California Cognitive Assessment Battery (CCAB). Frontiers in Human Neuroscience, 17, 1305529.